# Supplementary material for: Deep neural networks and humans both benefit from compositional language structure
Source: Nat Commun. 2024 Dec 30;15:10816. doi: 10.1038/s41467-024-55158-1 (PMC11685529; doi:10.1038/s41467-024-55158-1)
Supplement: Supplementary file 1 — Supplementary Information [file 41467_2024_55158_MOESM1_ESM.pdf]

Supplementary Information: Deep neural networks and humans both benefit from compositional language structure

Lukas Galke[1,2,✉], Yoav Ram[3,4], Limor Raviv[2,5]

[1]Department of Mathematics and Computer Science, University of Southern Denmark, Odense, Denmark [2]LEADS group, Max Planck Institute for Psycholinguistics, Nijmegen, Netherlands [3]School of Zoology, Faculty of Life Sciences, Tel Aviv University, Tel Aviv, Israel [4]Sagol School of Neuroscience, Tel Aviv University, Tel Aviv, Israel [5]cSCAN, University of Glasgow, Glasgow, UK [✉]email: galke@imada.sdu.dk

**Input Language**	**Structure Score**	**Ambiguity %**	**Structure Bin**
S1	0.09	0	1
B1	0.07	0	1
S2	0.25	0.35	2
B2	0.35	0.09	2
S3	0.59	0.13	3
B3	0.58	0.17	3
S4	0.79	0	4
B4	0.69	0	4
S5	0.84	0	5
B5	0.85	0	5

Supplementary Table 1: Structure scores of the input languages and categorization into bins, as well as the percentage of ambiguous words in the language (words referring to more than one meaning).

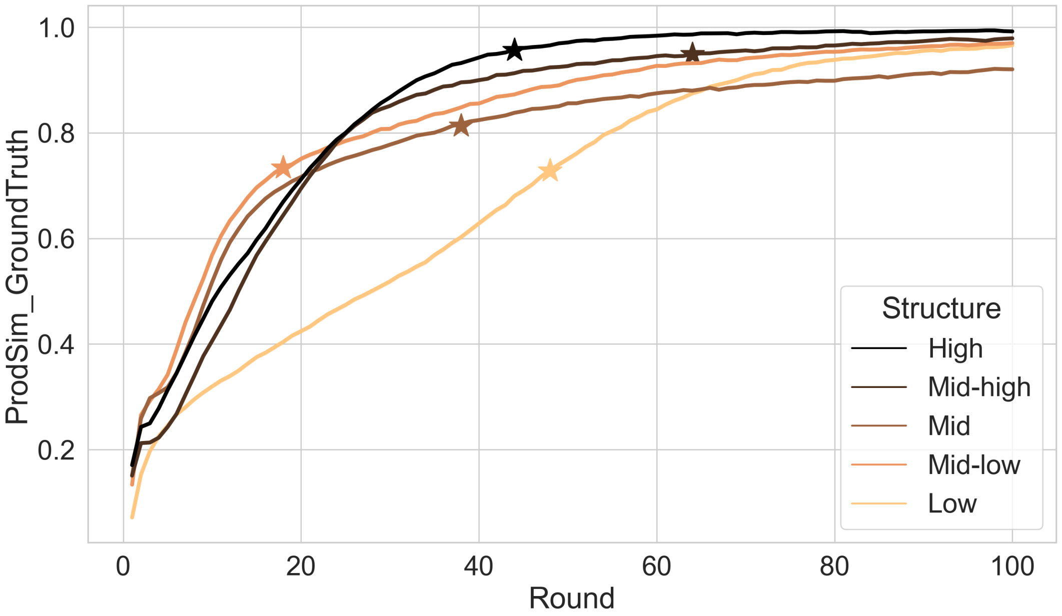

Supplementary Figure 1: Production Similarity to the ground truth of the input language as a function of round number. Color indicates the degree of structure (darker means higher). Stars indicate where neural network agents exceed human performance.

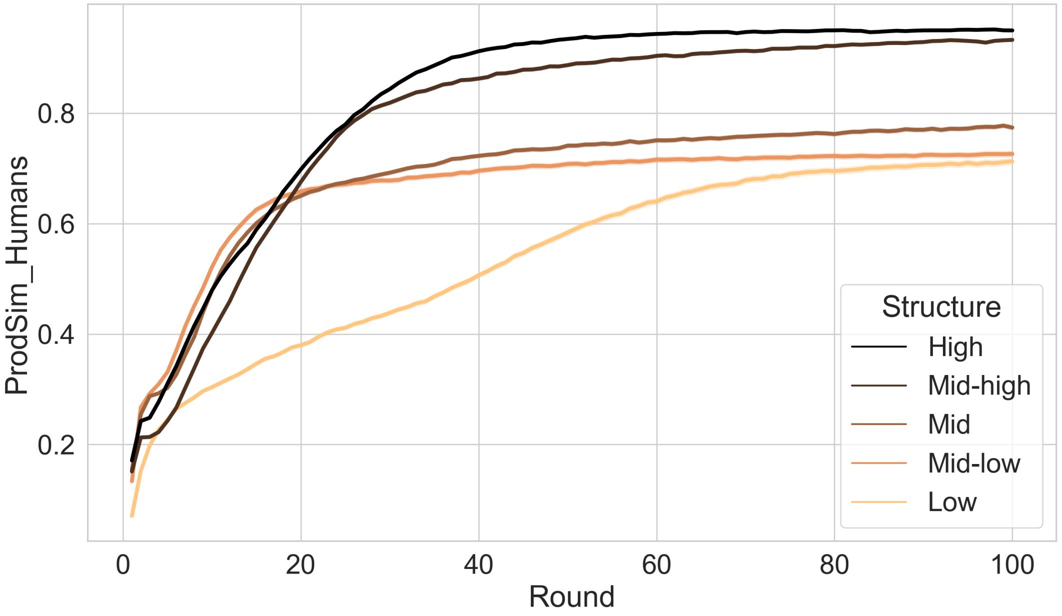

Supplementary Figure 2: Production similarity to humans learning the same input language during memorization. Color indicates the degree of structure (darker means higher).

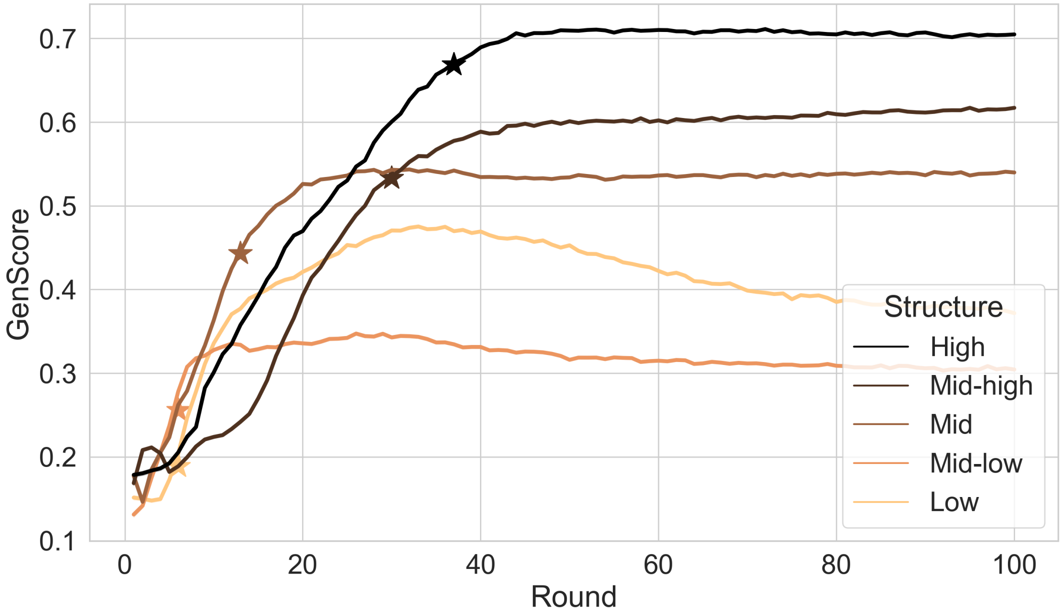

Supplementary Figure 3: Generalization score as a function of round number. Color represents the degree of structure. Stars indicate where neural network agents exceed human performance.

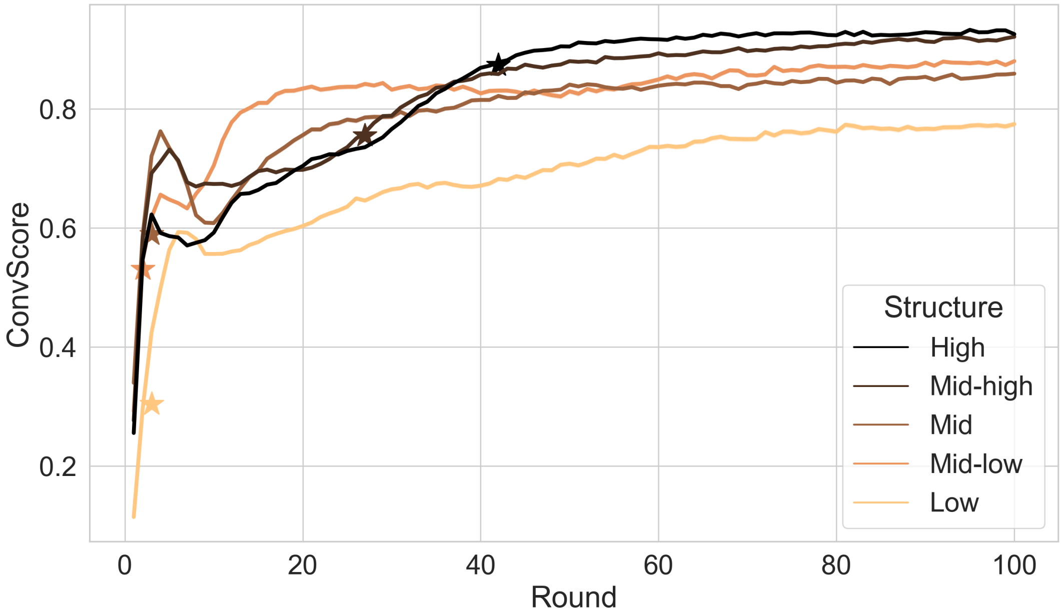

Supplementary Figure 4: Convergence score as a function of round number. Color indicates the degree of structure (darker means higher). Stars indicate where neural network agents exceed human performance.

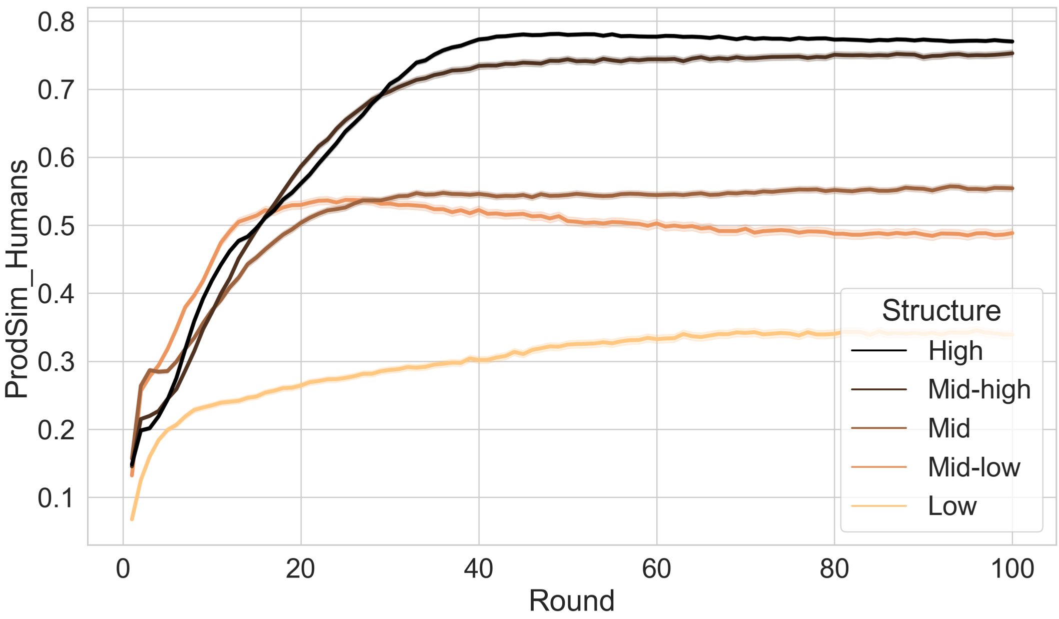

Supplementary Figure 5: Production Similarity to humans during generalization. Color indicates the degree of structure (darker means higher).

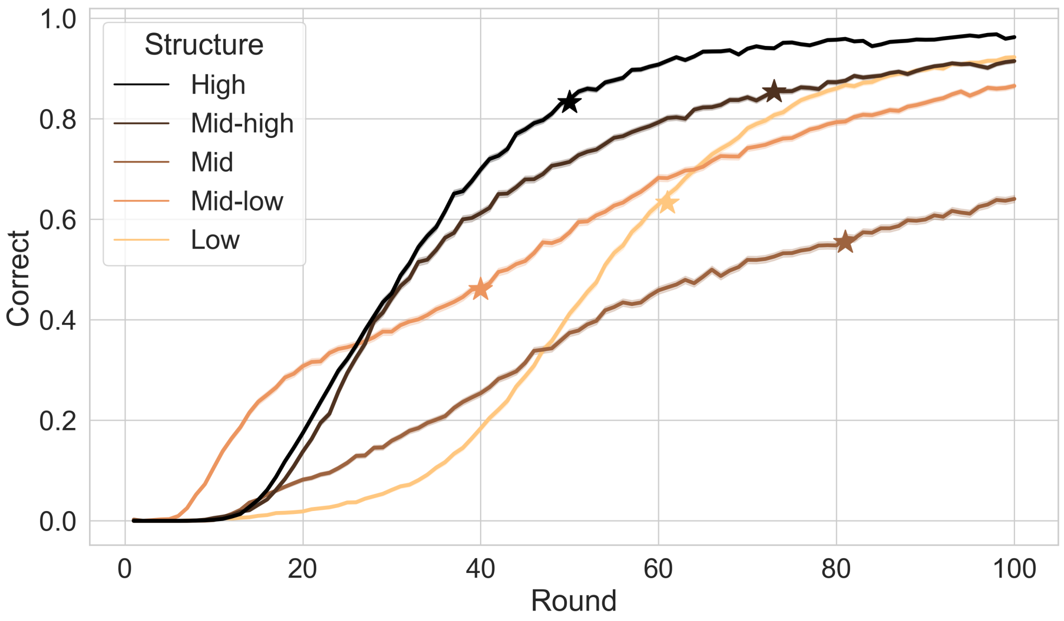

Supplementary Figure 6: Binary Accuracy with respect to ground truth of input languages as a function of round number. To compute binary accuracy, we compare the labels produced by the neural agents with the ground truth label of the input language and each label receives a score of one if it is exactly the same as the ground truth and zero otherwise. These boolean scores are then averaged to obtain binary accuracy. Color indicates the degree of structure (darker means higher). Stars indicate where neural network agents exceed human performance.

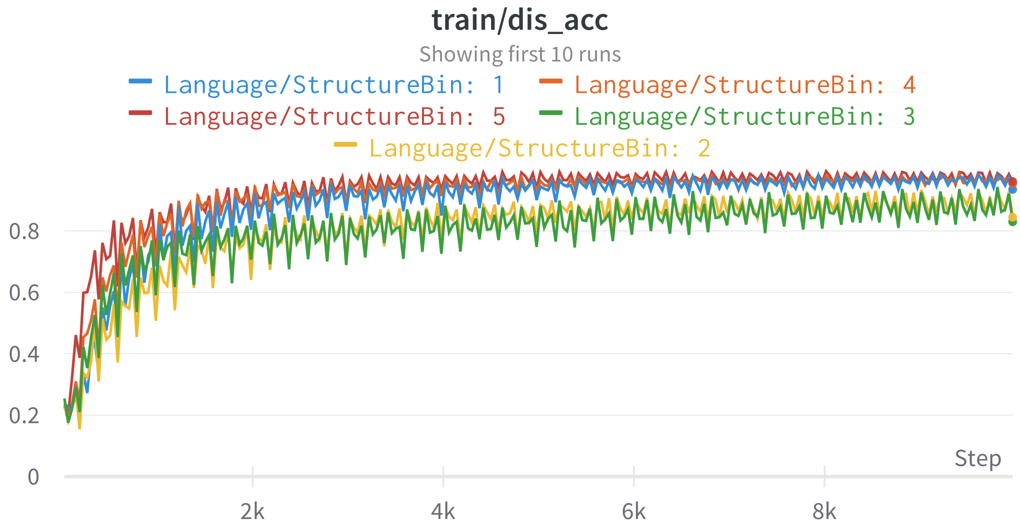

Supplementary Figure 7: Guessing Accuracy: selecting the right scene among distractors within the contrastive training objective during training. Guessing accuracy was calculated during training with active dropout since it was part of the training phase.

Supplementary Table 2: Linear Mixed-Effects Regression Results. LME 1: Production similarity to ground truth during memorization. LME 2: Production similarity to Humans during Generalization. LME 3: Generalization score. LME 4: Convergence score during generalization. LME 5: Production similarity to humans during generalization. All tests are two-sided.

**LME 1**: Production Similarity to Ground Truth	Coef.	Std.Err.	z	P> |z|	[0.025	0.975]
Intercept	0.797	0.001	1110.436	< 0.001	0.796	0.798
scale(StructureScore)	0.045	0.001	62.865	< 0.001	0.044	0.047
scale(np.log(Round))	0.199	< 0.001	2060.282	< 0.001	0.199	0.199
scale(StructureScore):scale(np.log(Round))	-0.005	< 0.001	-54.978	< 0.001	-0.005	-0.005
**LME 2**: Prod. Sim. to Humans during Memorization	Coef.	Std.Err.	z	P> |z|	[0.025	0.975]
Intercept	0.701	0.001	517.888	< 0.001	0.698	0.704
scale(StructureScore)	0.097	0.001	71.429	< 0.001	0.094	0.099
scale(np.log(Round))	0.156	< 0.001	1504.189	< 0.001	0.155	0.156
scale(StructureScore):scale(np.log(Round))	0.022	< 0.001	208.708	< 0.001	0.021	0.022
**LME 3**: Generalization Score	Coef.	Std.Err.	z	P> |z|	[0.025	0.975]
Intercept	0.468	0.001	790.838	< 0.001	0.467	0.469
scale(StructureScore)	0.088	0.001	148.901	< 0.001	0.087	0.089
scale(np.log(Round))	0.084	< 0.001	1281.568	< 0.001	0.084	0.084
scale(StructureScore):scale(np.log(Round))	0.046	< 0.001	703.483	< 0.001	0.046	0.046
**LME 4**: Convergence Score during Generalization	Coef.	Std.Err.	z	P> |z|	[0.025	0.975]
Intercept	0.792	0.001	900.121	< 0.001	0.790	0.794
scale(StructureScore)	0.043	0.001	49.027	< 0.001	0.041	0.045
scale(np.log(Round))	0.094	< 0.001	1220.090	< 0.001	0.094	0.094
scale(StructureScore):scale(np.log(Round))	0.009	< 0.001	121.740	< 0.001	0.009	0.010
**LME 5**: Prod. Sim. to Humans during Generalization	Coef.	Std.Err.	z	P> |z|	[0.025	0.975]
Intercept	0.529	0.002	280.903	< 0.001	0.525	0.533
scale(StructureScore)	0.132	0.002	70.280	< 0.001	0.129	0.136
center(np.log(Round))	0.101	< 0.001	749.746	< 0.001	0.101	0.101
scale(StructureScore):center(np.log(Round))	0.046	< 0.001	344.287	< 0.001	0.046	0.047

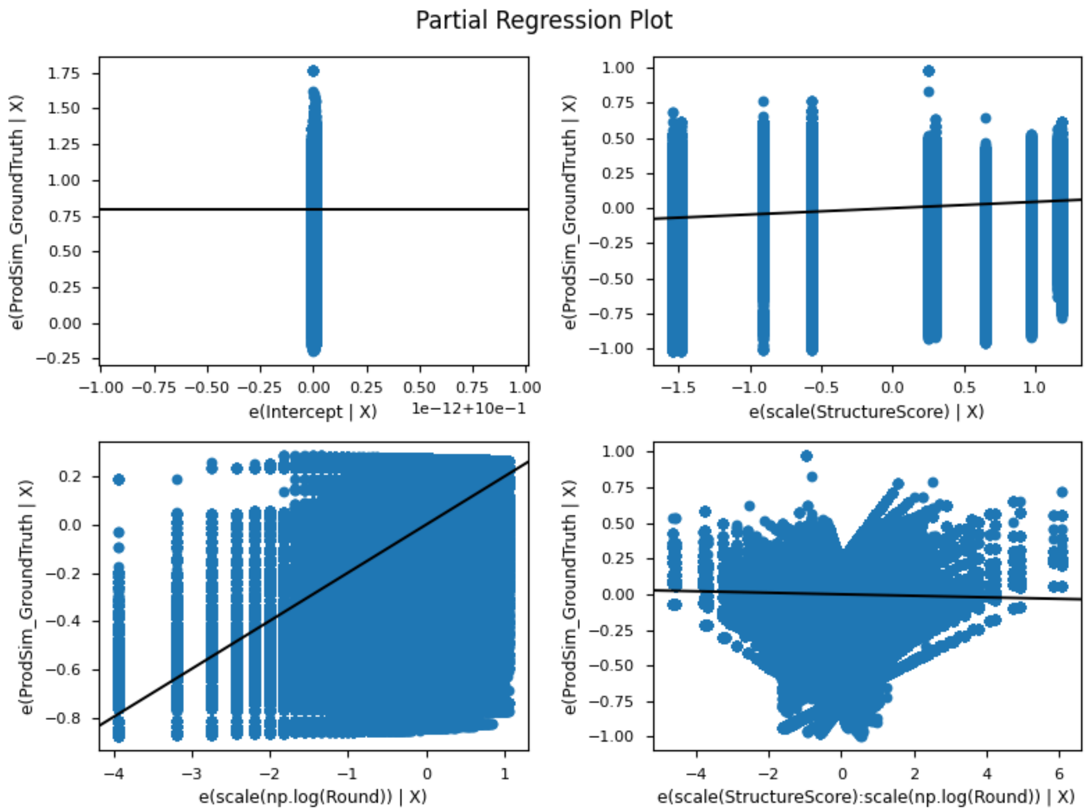

Supplementary Figure 8: Partial regression plots of LME 1: Production Similarity to ground truth during memorization

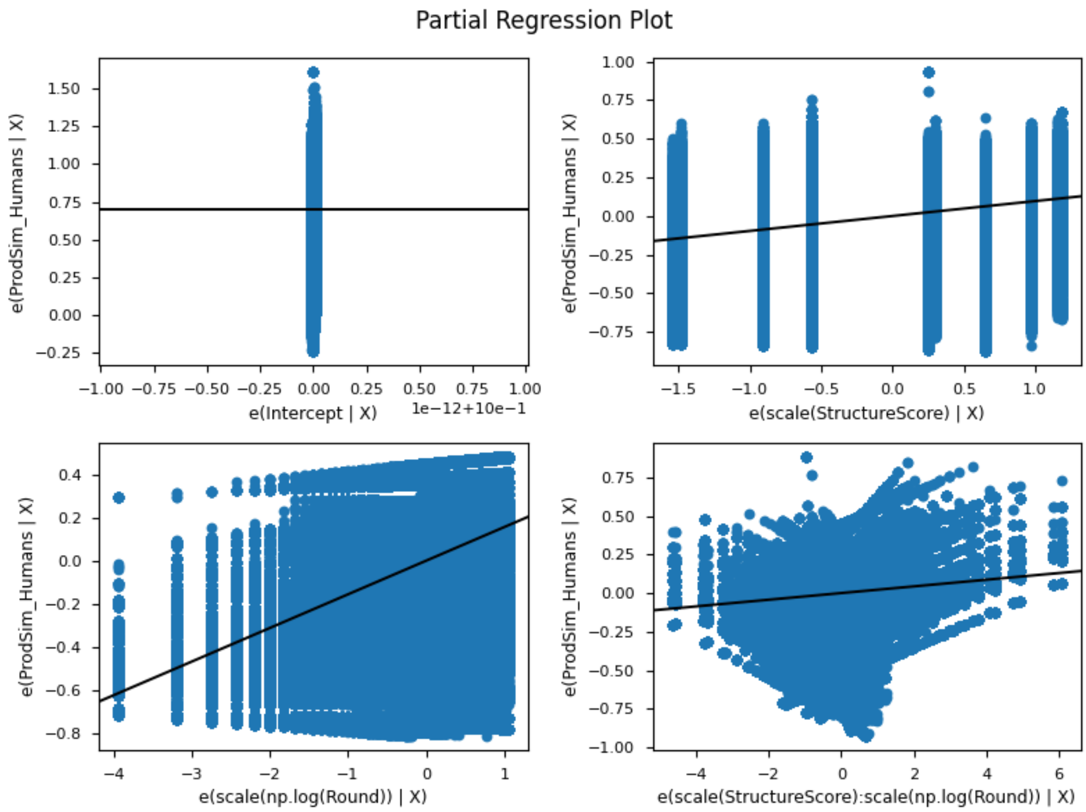

Supplementary Figure 9: Partial regression plots of LME 1: Production Similarity to humans during Memorization

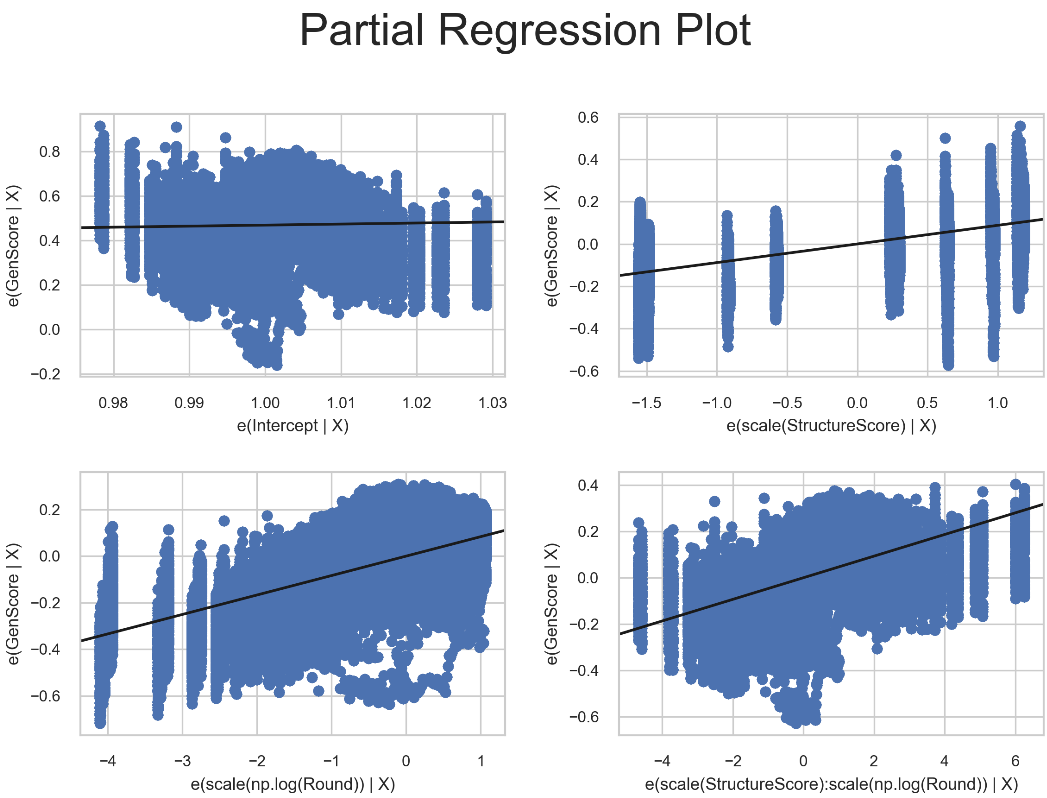

Supplementary Figure 10: Partial regression plots of LME 3: Generalization Score

Supplementary Table 3: Linear Mixed-Effects Regression Results of LME 6: Production similarity to humans during memorization at a fixed round number. All tests are two-sided.

**Round 10**	Coef.	Std.Err.	z	P> |z|	[0.025	0.975]
Intercept	0.459	< 0.001	1009.015	< 0.001	0.458	0.459
scale(StructureScore)	0.024	0.001	16.470	< 0.001	0.021	0.027
**Round 40**	Coef.	Std.Err.	z	P> |z|	[0.025	0.975]
Intercept	0.830	< 0.001	2370.225	< 0.001	0.829	0.831
scale(StructureScore)	0.094	0.001	78.439	< 0.001	0.091	0.096
**Round 70**	Coef.	Std.Err.	z	P> |z|	[0.025	0.975]
Intercept	0.936	< 0.001	19666.003	< 0.001	0.936	0.936
scale(StructureScore)	0.021	0.001	23.322	< 0.001	0.020	0.023
**Round 100**	Coef.	Std.Err.	z	P> |z|	[0.025	0.975]
Intercept	0.965	< 0.001	31871.611	< 0.001	0.965	0.965
scale(StructureScore)	0.005	0.001	7.725	< 0.001	0.004	0.007

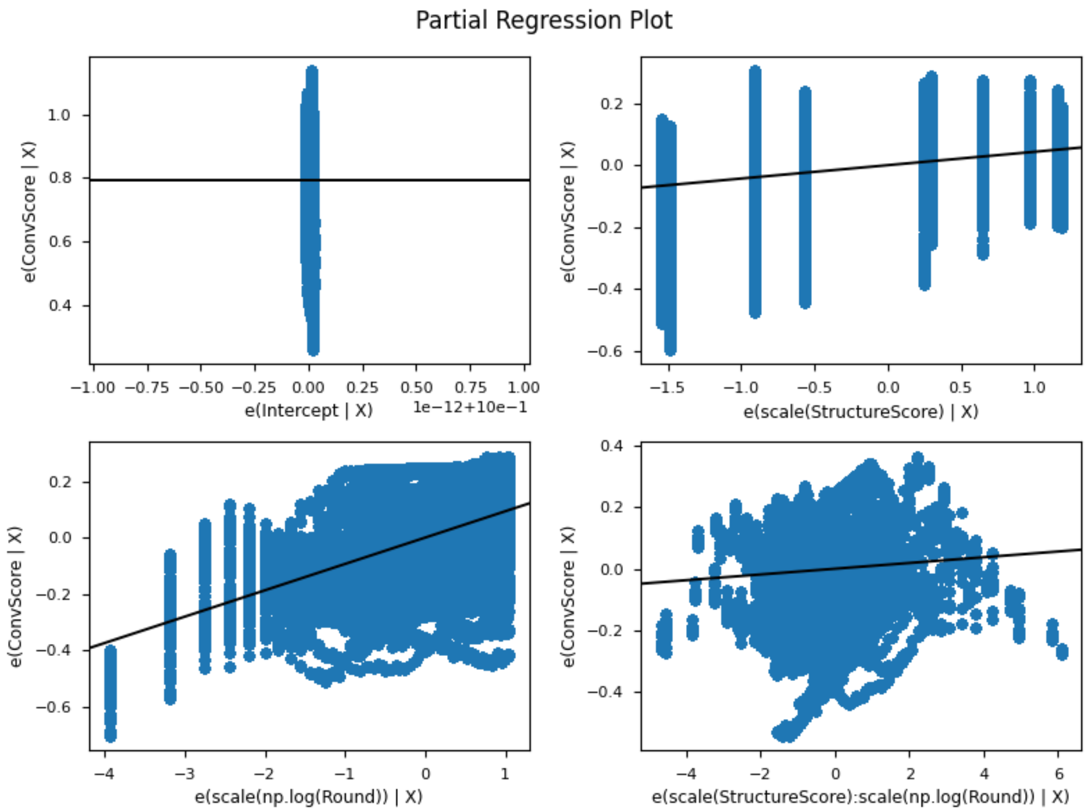

Supplementary Figure 11: Partial regression plots of LME 4: Convergence Score (during Generlization)

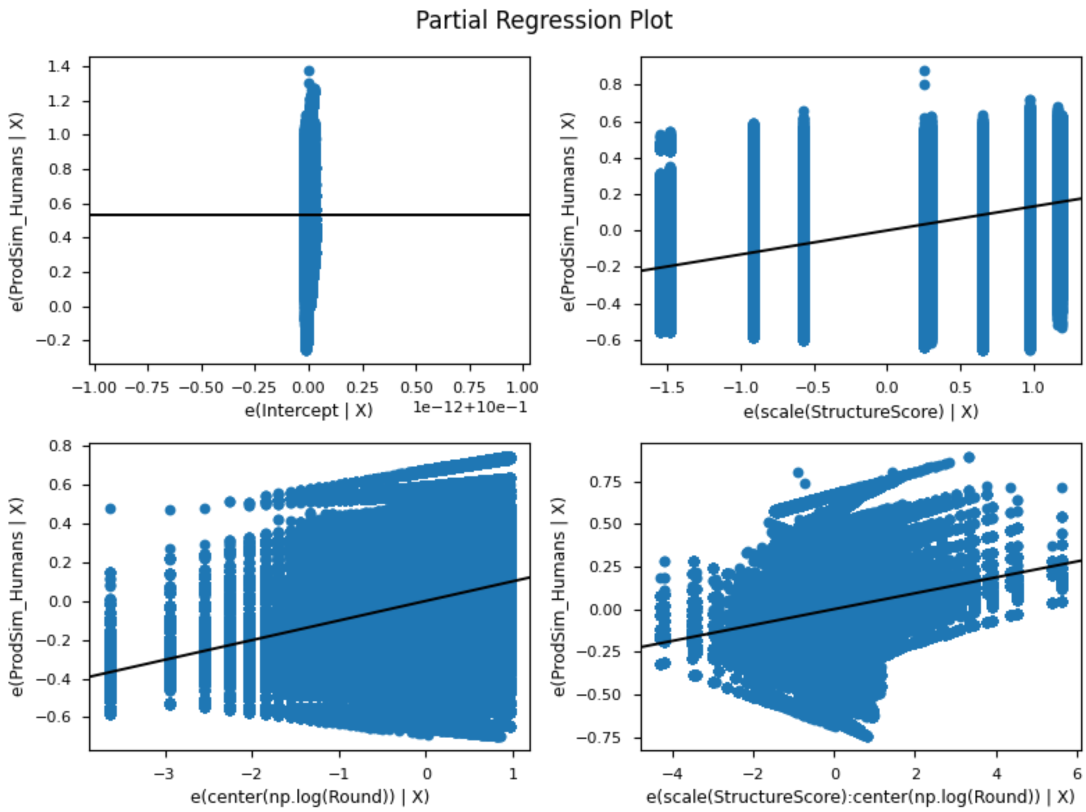

Supplementary Figure 12: Partial regression plots of LME 5: Production similarity to humans during generalization

Supplementary Methods

We found the training robust to most experimental configurations, such as learning rate, number of layers, and whether the parameters are shared between reader and writer models. However, one particular hyperparameter that affects the model capacity has a substantial effect on the learning speed: the size of the hidden layers, for which we provide a sensitivity analysis in the following. Subsequently, we also provide a sensitivity analysis of the scaling factor α for the contrastive loss term $\mathcal{L}_{\text{con}}$.

Sensitivity to Hidden Layer Size

We vary the hidden size and plot the average scores over the 10 input languages. Supplementary Figure 13 shows the production similarity during memorization. Supplementary Figure 14 shows the production similarity between neural agents and human learners during testing. Supplementary Figure 15 shows the generalization score of neural agents.

Sensitivity to the Scaling Factor for the Contrastive Loss Term

We experiment with different scaling factors α for the contrastive loss term. Supplementary Figure 16 shows the results for production similarity with ground truth during the memorization test. Here, we report the average across all input languages with different degrees of structuredness. Supplementary Figure 17 shows the results for production similarity with human learners during the generalization test. Again, a scaling factor of 0.1 leads to the best results in terms of learning speed. However, there is little difference to using a scaling factor of 0.2. Supplementary Figure 18 shows the results for generalization score (scaled to $[0, 1]$). We observe that the scaling factor of 0.1 has advantages in learning speed. Starting at step 1,300, the generalization score increases faster with scaling factor 0.1 than with other scaling factors.

Structure effect does not depend on specific hyperparameter choices

Supplementary Figure 19 and Supplementary Figure 20 show the relationship between the degree of compositional structure and the generalizations core with respect to the hidden size and the scaling factor for the contrastive loss.

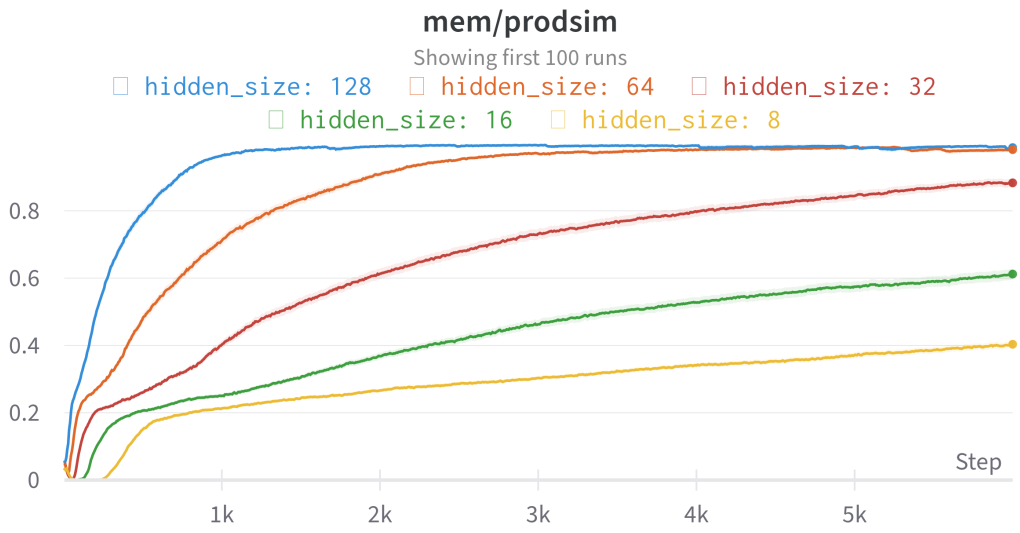

Supplementary Figure 13: Average production similarity to ground truth during memorization across input languages as a function of the size of the neural networks' hidden layers.

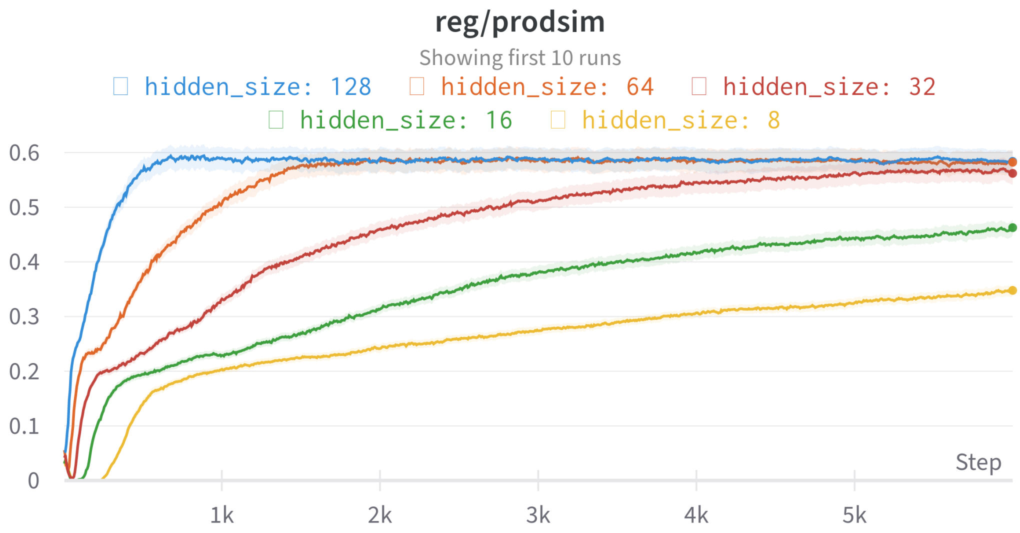

Supplementary Figure 14: Average production similarity to humans across input languages as a function of the size of the neural networks' hidden layers.

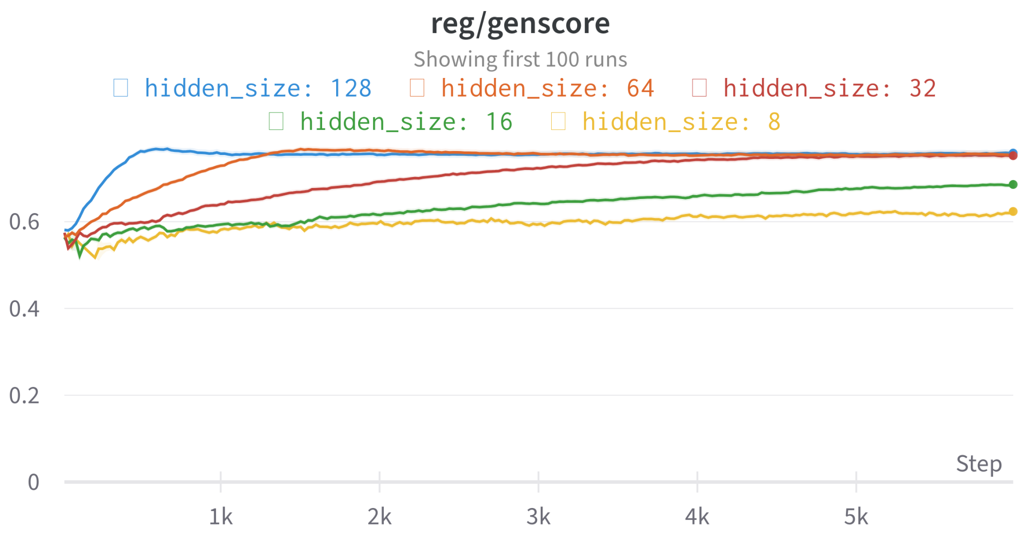

Supplementary Figure 15: Average generalization systematicity across input languages as a function of the size of the neural networks' hidden layers

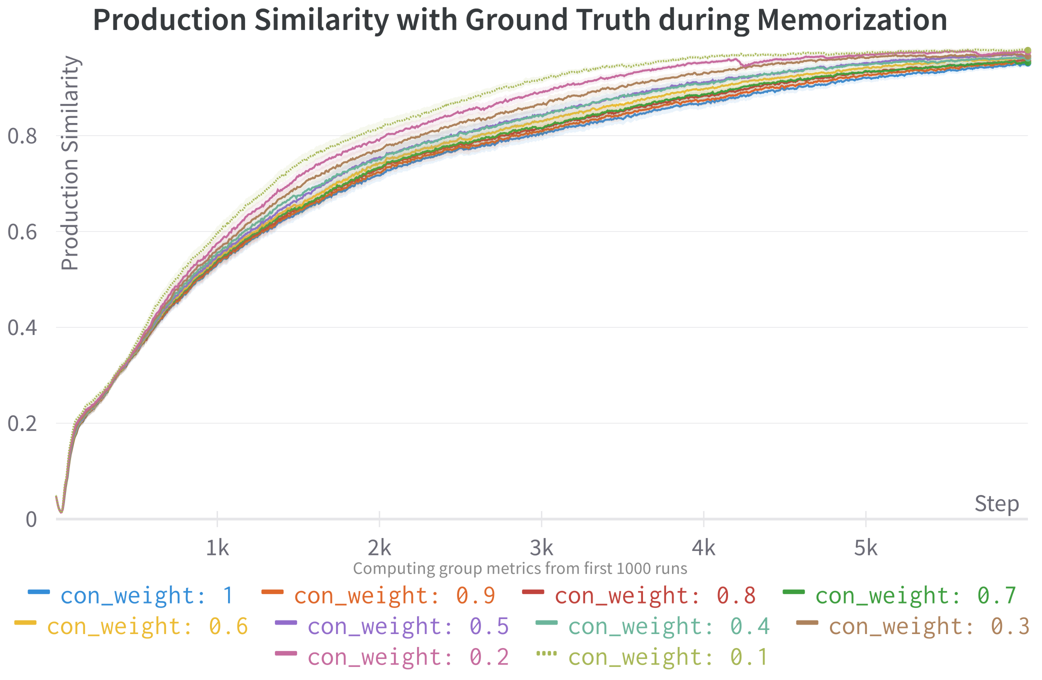

Supplementary Figure 16: Average production similarity to ground truth across input languages during memorization as a function of the scaling factor for the contrastive loss term.

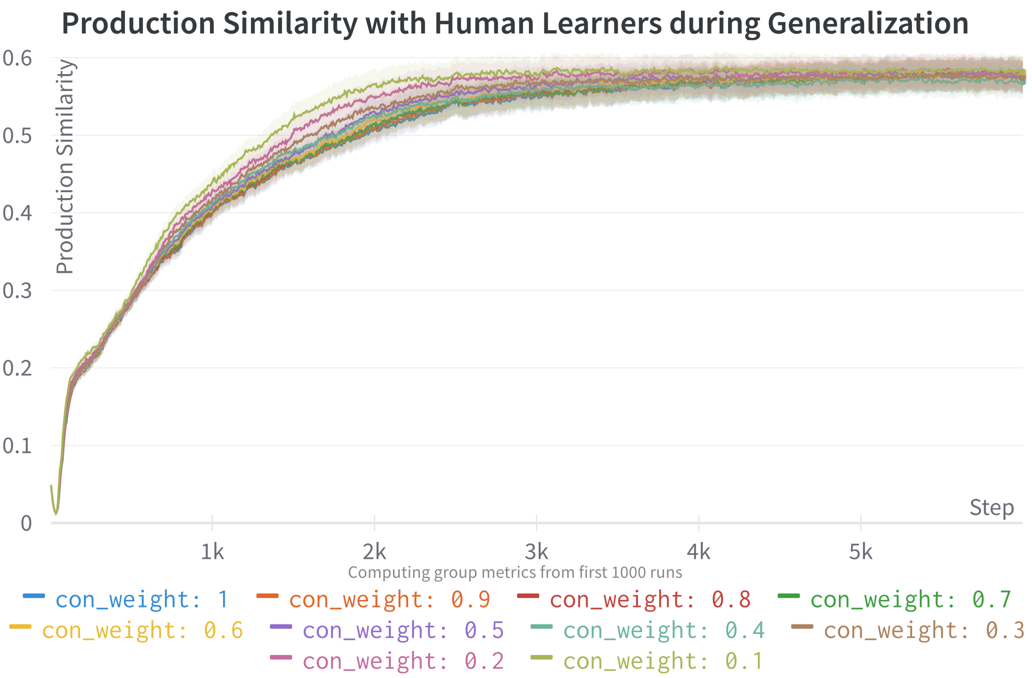

Supplementary Figure 17: Average production similarity with human learners across input languages during generalization as a function of the scaling factor for the contrastive loss term.

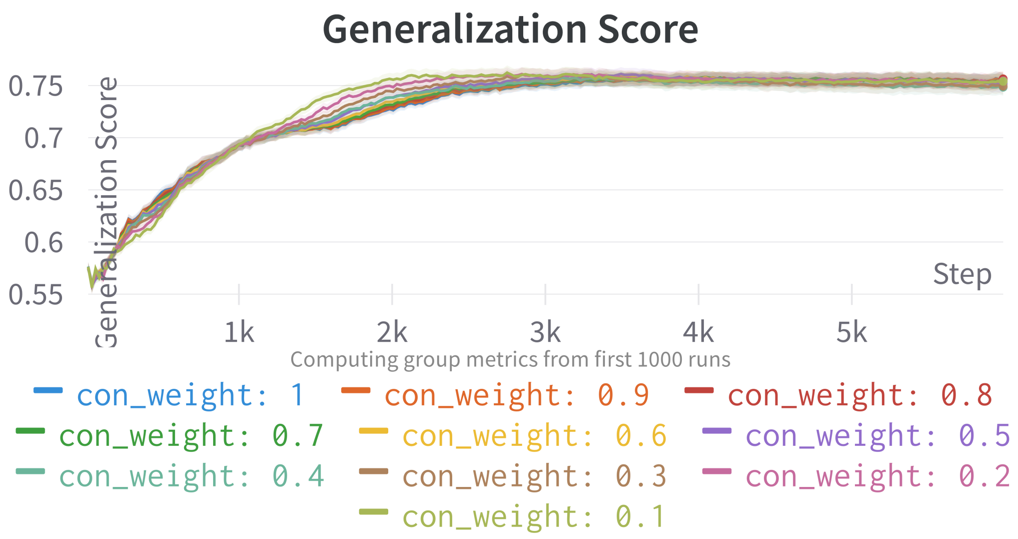

Supplementary Figure 18: Average generalization score across input languages as a function of the scaling factor for the contrastive loss term.

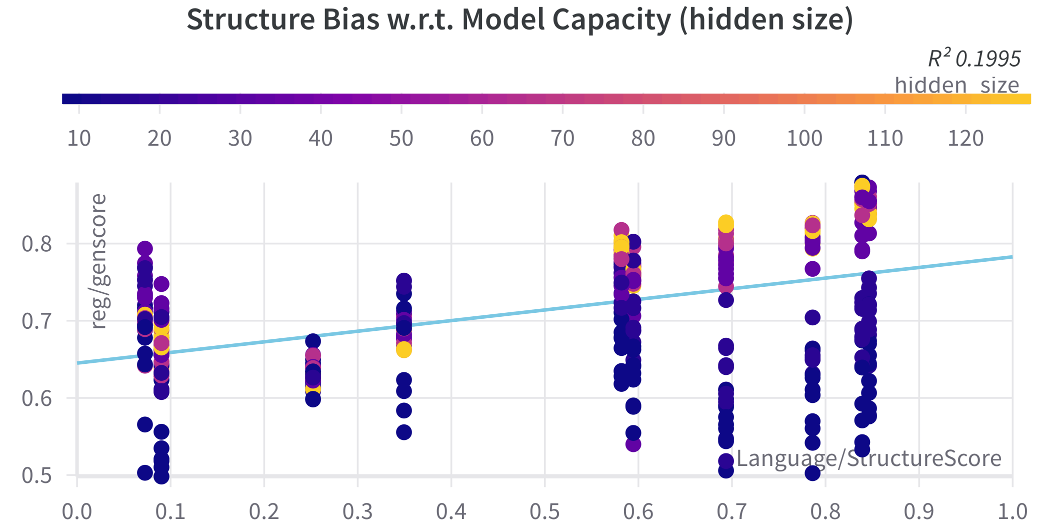

Supplementary Figure 19: Relationship between compositional structure and generalization score with different values for hidden size controlling the model capacity

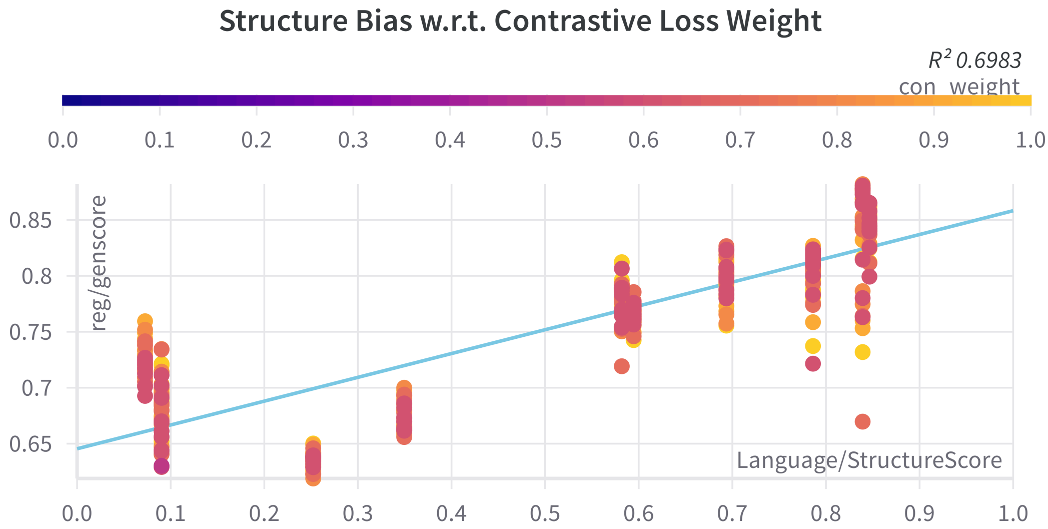

Supplementary Figure 20: Relationship between compositional structure and generalization score with different values for contrastive loss weight controlling the influence of the contrastive objective used for guessing tasks

Supplementary Table 4: Example Data from the Memorization Test. The sample is stratified with respect to the by-producer average production similarity to human participants. For each percentile out of 0, 25, 50, 75, and 100, we randomly sample 8 items. All samples are taken at the end of training (after epoch 100).

Sim. to Humans	Producer	Lang.	Shape	Angle	True Label	Participant Label	RNN Label
0.247826	4082	S1	4	225	wufk	moof	wufk
0.247826	4082	S1	2	330	weft	puif	weft
0.247826	4082	S1	1	240	smoogg	huift	smoogg
0.247826	4082	S1	4	300	suith	keft	suith
0.247826	4082	S1	3	135	spuut	weft	spuut
0.247826	4082	S1	1	60	koof	puif	koof
0.247826	4082	S1	4	120	wafk	sgeft	wafk
0.247826	4082	S1	3	270	huipt	koom	huipt
0.700932	1093	B2	4	270	gnt	gnt	gnt
0.700932	1093	B2	4	135	gnts	gntsi	gnts
0.700932	1093	B2	2	330	gmps	gmps	gmps
0.700932	1093	B2	2	120	wangsus	wangsus	wangsu
0.700932	1093	B2	2	45	gempt	wangsus	gempt
0.700932	1093	B2	4	330	gntsi	gnit	gntsi
0.700932	1093	B2	2	300	wangsu	gmps	wangsu
0.700932	1093	B2	1	30	sket	sket	sket
0.883282	3090	S5	2	180	nif-a	nif-a	nif-as
0.883282	3090	S5	1	90	wef-t	wef-t	wef-t
0.883282	3090	S5	1	150	wef-aat	wef-att	wef-aat
0.883282	3090	S5	1	240	wef-ssa	wef-aas	wef-ssa
0.883282	3090	S5	4	210	smut-aas	smut-aas	smut-aas
0.883282	3090	S5	3	180	pti-a	pti-a	pti-a
0.883282	3090	S5	3	315	pti-kks	pti-ssk	pti-kks
0.883282	3090	S5	2	225	nif-as	nif-as	nif-as
0.956522	1020	B1	2	330	wak-ta	wak-ta	wak-ta
0.956522	1020	B1	4	225	gtgt	gtgt	gtgt
0.956522	1020	B1	4	120	ftft	ftft	ftft
0.956522	1020	B1	2	150	hehi	hehi	hehi
0.956522	1020	B1	1	210	ha-ia	ha-ia	ha-ia
0.956522	1020	B1	3	45	fiti	fiti	fiti
0.956522	1020	B1	4	60	kite	fik	kite
0.956522	1020	B1	4	360	pepepe	pepepe	pepepe
1.000000	1008	B4	4	225	fak-huif	fak-huif	fak-huif
1.000000	1008	B4	3	45	muif-a	muif-a	muif-a
1.000000	1008	B4	4	120	fak-e	fak-e	fak-e
1.000000	1008	B4	1	315	fas-pok	fas-pok	fas-pok
1.000000	1008	B4	3	135	muif-e	muif-e	muif-e
1.000000	1008	B4	2	135	pok-e	pok-e	pok-e
1.000000	1008	B4	4	300	fak-pok	fak-pok	fak-pok
1.000000	1008	B4	2	210	pok-huif	pok-huif	pok-huif

Supplementary Table 5: Example Data from the Generalization Test. The sample is stratified with respect to the by-producer average production similarity to human participants. For each percentile out of 0, 25, 50, 75, and 100, we randomly sample 8 items. All samples are taken at the end of training (after epoch 100).

Sim. to Humans	Producer	Lang.	Shape	Angle	Participant Label	RNN Label
0.092308	1048	B1	2	360	kokoke	seefe
0.092308	1048	B1	1	225	po-ti	ha-ia
0.092308	1048	B1	3	90	ghio	mimi
0.092308	1048	B1	4	240	khio	gtgt
0.092308	1048	B1	3	150	ptpt	mimi
0.092308	1048	B1	2	300	ko-toe	wak-ta
0.092308	1048	B1	4	210	ka-ia	gtgt
0.092308	1048	B1	2	225	haia	pooti
0.443223	8064	B2	3	150	wangsi	wangsuu
0.443223	8064	B2	4	225	gntsoe	gntuu
0.443223	8064	B2	1	135	sketsi	gesh
0.443223	8064	B2	4	360	gnt	skek
0.443223	8064	B2	4	60	gmpsi	skek
0.443223	8064	B2	2	270	wng	wangsuu
0.443223	8064	B2	2	360	wang	gempt
0.443223	8064	B2	3	225	wangsuus	wangsoe
0.590884	1083	S3	4	60	fuottee	fuoto-o-o-o
0.590884	1083	S3	4	150	fuottoo	fuottii
0.590884	1083	S3	1	30	fewo-o-o-o	fewen
0.590884	1083	S3	3	60	powi	powu-u-u
0.590884	1083	S3	1	225	fewo-o-o-o	fewo-o-o
0.590884	1083	S3	3	225	powee	powwoo
0.590884	1083	S3	2	360	asken	asko-o-o
0.590884	1083	S3	4	330	fuottoa	fuotio
0.772497	2058	B4	2	360	pok-i	pok
0.772497	2058	B4	4	330	fak-pok	fas-i
0.772497	2058	B4	4	60	fak-a	fak-e
0.772497	2058	B4	1	30	fas-a	fas-a
0.772497	2058	B4	2	300	pok	pok-u
0.772497	2058	B4	4	90	fak-u	fak-e
0.772497	2058	B4	4	150	fak-w-w-e	fak-e
0.772497	2058	B4	3	225	muif-huif	muif-huif
0.923443	5016	B4	4	90	fak-iii	fak-e
0.923443	5016	B4	1	120	fas-e	fas-e
0.923443	5016	B4	2	300	pok	pok
0.923443	5016	B4	3	60	muif-a	muif-a
0.923443	5016	B4	3	360	muif-i	muif-a
0.923443	5016	B4	4	150	fak-e	fak-e
0.923443	5016	B4	3	225	muif-huif	muif-huif
0.923443	5016	B4	4	150	fak-e	fak-e
