# Supplementary material for: Deep neural networks and humans both benefit from compositional language structure
Source: Nat Commun. 2024 Dec 30;15:10816. doi: 10.1038/s41467-024-55158-1 (PMC11685529; doi:10.1038/s41467-024-55158-1)
Supplement: Supplementary file 2 — Reporting Summary [file 41467_2024_55158_MOESM2_ESM.pdf]

nature portfolio

Corresponding author(s): Lukas Galke

Last updated by author(s): 10/25/2024

Reporting Summary

Nature Portfolio wishes to improve the reproducibility of the work that we publish. This form provides structure for consistency and transparency in reporting. For further information on Nature Portfolio policies, see our Editorial Policies and the Editorial Policy Checklist.

Statistics

For all statistical analyses, confirm that the following items are present in the figure legend, table legend, main text, or Methods section.

n/a	Confirmed	
☐	☒	The exact sample size (*n*) for each experimental group/condition, given as a discrete number and unit of measurement
☐	☒	A statement on whether measurements were taken from distinct samples or whether the same sample was measured repeatedly
☐	☒	The statistical test(s) used AND whether they are one- or two-sided<br>*Only common tests should be described solely by name; describe more complex techniques in the Methods section.*
☐	☒	A description of all covariates tested
☐	☒	A description of any assumptions or corrections, such as tests of normality and adjustment for multiple comparisons
☐	☒	A full description of the statistical parameters including central tendency (e.g. means) or other basic estimates (e.g. regression coefficient) AND variation (e.g. standard deviation) or associated estimates of uncertainty (e.g. confidence intervals)
☐	☒	For null hypothesis testing, the test statistic (e.g. *F*, *t*, *r*) with confidence intervals, effect sizes, degrees of freedom and *P* value noted<br>*Give P values as exact values whenever suitable.*
☒	☐	For Bayesian analysis, information on the choice of priors and Markov chain Monte Carlo settings
☒	☐	For hierarchical and complex designs, identification of the appropriate level for tests and full reporting of outcomes
☒	☐	Estimates of effect sizes (e.g. Cohen's *d*, Pearson's *r*), indicating how they were calculated

Our web collection on statistics for biologists contains articles on many of the points above.

Software and code

Policy information about availability of computer code

Data collection	Machine learning agents were implemented with PyTorch and the experimental setup is implemented in Python. Code with all details is available: https://github.com/lgalke/easy2deeplearn
Data analysis	Data analysis was performed with the Python package Statsmodels and visualized via Seaborn. Code with all details is available: Machine learning agents were implemented with PyTorch and the experimental setup is implemented in Python. Code with all details is available: https://github.com/lgalke/easy2deeplearn

For manuscripts utilizing custom algorithms or software that are central to the research but not yet described in published literature, software must be made available to editors and reviewers. We strongly encourage code deposition in a community repository (e.g. GitHub). See the Nature Portfolio guidelines for submitting code & software for further information.

Data

Policy information about availability of data

All manuscripts must include a data availability statement. This statement should provide the following information, where applicable:

- Accession codes, unique identifiers, or web links for publicly available datasets
- A description of any restrictions on data availability
- For clinical datasets or third party data, please ensure that the statement adheres to our policy

The data from our experiments is made available via FigShare and linked/cited in the main text.

Research involving human participants, their data, or biological material

Policy information about studies with human participants or human data. See also policy information about sex, gender (identity/presentation), and sexual orientation and race, ethnicity and racism.

Reporting on sex and gender	No new data from human participants were generated in this study. Human data for comparison is taken from: Limor Raviv, Marianne de Heer Kloots, Antje Meyer, What makes a language easy to learn? A preregistered study on how systema)c structure and community size affect language learnability, Cognition, Volume 210, 2021, 104620, ISSN 0010-0277, https://doi.org/10.1016/j.cognition.2021.104620<br><br>The dataset contains data from 100 participants, out of which 79 were female.
Reporting on race, ethnicity, or other socially relevant groupings	No new data from human participants were generated in this study. Human data for comparison is taken from: Limor Raviv, Marianne de Heer Kloots, Antje Meyer, What makes a language easy to learn? A preregistered study on how systema)c structure and community size affect language learnability, Cognition, Volume 210, 2021, 104620, ISSN 0010-0277, https://doi.org/10.1016/j.cognition.2021.104620<br><br>All participants were native Durch speakers and had no reported visual or reading difficulties
Population characteristics	No new data from human participants were generated in this study. Human data for comparison is taken from: Limor Raviv, Marianne de Heer Kloots, Antje Meyer, What makes a language easy to learn? A preregistered study on how systema)c structure and community size affect language learnability, Cognition, Volume 210, 2021, 104620, ISSN 0010-0277, https://doi.org/10.1016/j.cognition.2021.104620<br><br>The mean age in that study was 22.9y
Recruitment	No new data from human participants were generated in this study. Human data for comparison is taken from: Limor Raviv, Marianne de Heer Kloots, Antje Meyer, What makes a language easy to learn? A preregistered study on how systema)c structure and community size affect language learnability, Cognition, Volume 210, 2021, 104620, ISSN 0010-0277, https://doi.org/10.1016/j.cognition.2021.104620
Ethics oversight	No new data from human participants were generated in this study. Human data for comparison is taken from: Limor Raviv, Marianne de Heer Kloots, Antje Meyer, What makes a language easy to learn? A preregistered study on how systema)c structure and community size affect language learnability, Cognition, Volume 210, 2021, 104620, ISSN 0010-0277, https://doi.org/10.1016/j.cognition.2021.104620<br><br>Ethical approval for that study was granted by the Faculty of Social Sciences of Radboud University Nijmegen

Note that full information on the approval of the study protocol must also be provided in the manuscript.

Field-specific reporting

Please select the one below that is the best fit for your research. If you are not sure, read the appropriate sections before making your selection.

☐ Life sciences ☒ Behavioural & social sciences ☐ Ecological, evolutionary & environmental sciences

For a reference copy of the document with all sections, see nature.com/documents/nr-reporting-summary-flat.pdf

Behavioural & social sciences study design

All studies must disclose on these points even when the disclosure is negative.

Study description	A quantitative comparison of neural network models with previous data from human participants. We test computational models (deep neural networks) on artificial language learning tasks. We compare their results with data from humans. The data from humans comes from a previous study: Raviv, de Heer Kloots, A Meyer (2021): What makes a language easy to learn? A pre-registered study on how systematic structure and community size affect language learnability. Cognition 210.
Research sample	N/A
Sampling strategy	N/A
Data collection	N/A
Timing	N/A
Data exclusions	N/A

Non-participation	N/A
Randomization	N/A

Reporting for specific materials, systems and methods

We require information from authors about some types of materials, experimental systems and methods used in many studies. Here, indicate whether each material, system or method listed is relevant to your study. If you are not sure if a list item applies to your research, read the appropriate section before selecting a response.

Materials & experimental systems

n/a	Involved in the study	
☒	☐	Antibodies
☒	☐	Eukaryotic cell lines
☒	☐	Palaeontology and archaeology
☒	☐	Animals and other organisms
☒	☐	Clinical data
☒	☐	Dual use research of concern
☒	☐	Plants

Methods

n/a	Involved in the study	
☒	☐	ChIP-seq
☒	☐	Flow cytometry
☒	☐	MRI-based neuroimaging

Plants

Seed stocks	N/A
Novel plant genotypes	N/A
Authentication	N/A
